# Biographical Sketch of the Late Josiah Trowbridge, M.D.

**Published:** 1869-07

**Authors:** John S. Trowbridge


### BIOGRAPHICAL SKETCH

OF THE LATE

### JOSIAH TROWBRIDGE, M. D.

OF BUFFALO.

WITH NOTICES OF HIS COTEMPORARIES BEFORE AND DURING THE WAR OF 1812, AND HIS COPARTNERS.

---

PREPARED BY DR. JOHN S. TROWBRIDGE,

AT THE REQUEST OF THE ERIE COUNTY MEDICAL SOCIETY.

---

BUFFALO:
THE COURIER COMPANY, PRINTERS.

1869

### BIOGRAPHICAL SKETCH.

*Mr. President and Gentlemen of the Erie County Medical Society:*

In a country, the government of which has had an existence of less than a century, and the history of which is nevertheless replete with incidents, the labors of the historian and biographer are by no means easy. Historical, political and social changes are so rapid and novel, and of such magnitude, that there can be but little more accomplished in this direction than the simple recording of events. It remains for the future to establish their philosophy and measure their ultimate results upon society, whether they be for good or for evil. Generally speaking, this scantiness of material for personal history, is more a matter of regret than surprise. It is too common a fact to excite astonishment.

But little is known of the personality of most of the early and eminent medical practitioners of this country. The lecturer and author have in many ways left material for biography. "Life is a story, it is a short story, but it is the whole story." It is a story that is told of the doers of great deeds, or the thinkers of great thoughts, who lived or died in the past, and anterior to the biographer who describes, or to the poet who immortalizes.

This is peculiarly applicable to the early medical men of our land. It was only late in life that they became the centers of observation, and in a period that we may call, not only historical, but recent. It is to be regretted that men who lived in the open light of day, and so continually associated with their fellows, should have left so deplorable a lack of material for biography to transmit to coming generations; but, drifting over this vast and comparatively unexplored country, as circumstances or their interest might dictate, they left scarcely any records of their doings.

Their uncertain wanderings were attended with experiences which, had they been preserved, would in many instances be instructive and curious, and, oftentimes savor highly of romance.

This peculiarity of the American character, so common to our noble progenitors, has been too little noticed, and, in this "fast age," only appreciated when recorded as part and parcel of our local history. Having their origin in that fruitful nursery of men—and great men, in the most enlarged sense of the word—their progress has been steadily westward until the sons of New England now largely populate every Northern State and Territory from the Atlantic to the Pacific, carrying the church, the school and the other noble influences of New England civilization with them. In this there is true romance, but of a character unlike that of the Crusades of earlier centuries. It is romance under the guidance of God. It is, in reality, preaching the Gospel to all nations.

The necessities and privations of those pioneer progressives were borne by the strong-minded and the brave sons of the older and more populous States. They came on foot, or on horseback, alone, or with a devoted wife, and perhaps young family, and so dared to plunge into the then unknown wilderness, inhabited by the savage, and, perhaps, worse than all, were compelled to encounter new forms of malarious disease from which none were exempt. Now the rail, steamboat, and other improved modes of travel, enable the emigrant to pass from one extreme to the other of our extended republic with rapidity, in safety and comfort.

I am led to dwell upon these reflections for the reason that this now prosperous and beautiful city and the country beyond, was, in the days of which I write, what now has scarcely an existence—an unexplored West. The truthful compass still points westward as it did then; but when we speak of American civilization, the East, the West, the North and the South, have lost their social, and almost their political and geographical significance.

We are now with the early and the more recent trials and the irresistible advances of civilization, leaving the past to complete its history, and compelling the statesman and politician of these times to acknowledge the fact that we have a common country. This mighty, this unexampled, and, I may say, miraculous work has been accomplished in less time than was occupied in the erection of most of the English cathedrals. A nation of over thirty millions of people has sprung into existence almost within the memories of those who have so recently left us that the remembrances of their virtues are still fresh in our hearts.

Among the brave spirits that so fearlessly shared in these privations, there were no men who were so uniformly found in the front rank as the physicians. And in this advance of civilization no class of men were required to make so many and continued sacrifices as they. Distance without roads, streams without bridges, were no cause for refusal when the calls of humanity reached them.

Speaking from my early remembrances of the privations of an honored father, and referring incidentally to those of his associates, I necessarily mention all the medical men of this region until after the close of the war of 1812, and those with whom he was afterwards associated. They were few in number, differing as men differ, but all possessed of commendable qualities, and eminently fitted for the responsibilities of pioneer life. I refer particularly to the two Drs. Chapin—Daniel and Cyrenius—and to Ebenezer Johnson. As evidence that I am not indulging in exaggeration, it is only necessary to refer to the testimony of a few noble men and women, honored monuments of the past, and living witnesses of the present, who shared with them the perils and privations attending the settlement of a new country. Those names are, with them, household words; and when referred to, it is with veneration and respect, sufficient to convince us of the present age that those pioneer physicians were more than ordinary men, and deserving the remembrance of all who are enjoying the substantial fruits of their labors. They *were* extraordinary men, shining examples of heroic faith, of invincible courage, of generous self-sacrifice, of bold and untiring enterprise. Their lives afford models to the genius of the age and the race. They aided in transforming the forests into a prosperous city and the adjacent country into beautiful fields; or, in the language of the eloquent Bancroft, "they lived the life of the American people, walked in its light, reasoned with its reason, thought with its power of thought, felt the beatings of its mighty heart, and were in every way the children of nature, the children of the West, the children of America."

Prominent among them was Dr. Daniel Chapin, and the opportunity of rescuing from unmerited oblivion this name, affords me more than ordinary pleasure. He was a graduate of Yale College, a cultivated man, a skillful physician, and was an intimate and personal friend of my father, Dr. Trowbridge. I am only able to present a few prominent incidents connected with his life, for the reason that those with whom he was most intimately associated are either numbered with the dead or have removed to distant parts of our country. I am informed that he was born in Salisbury, Ct., and that he first practiced medicine in Kinderhook, and afterwards in East Bloomfield. Turner in his history of the "Phelps and Gorham Purchase," page 188, says: "Dr. Chapin was the early physician. He was the next representative of Ontario county in the Legislature after Gen. Israel Chapin." He states that he removed to Buffalo in 1805, and died there in 1835. This is an error, since he removed to this vicinity in 1807, never was a resident of the village of Buffalo, but resided on a farm a few miles distant, located on the present Main street, and now owned and occupied by Elam R. Jewett, Esq. He died in 1821, at about sixty years of age. His death was partly induced by the many and continued exposures incident to the practice of his profession in times when it required an amount of personal courage, self-denial and hardship but little understood by us of the present day.

As I have in the preparation of this paper had occasion to reflect upon the trials incident to the practice of our profession in the rude times that tried the very souls of men, I am free to confess that I am at a loss to know what were the incentives to encourage them to persevere, when so many easier avenues to wealth and prominence presented themselves. The merchant, mechanic and agriculturist, all had a pleasant future to stimulate them to action. That educated men, accustomed to the ordinary refinements and comforts of life, should be willing to devote their days to the practice of a profession continually demanding an amount of self-denial and sacrifice unequaled and unlike any other profession, or avocation, is to my mind beyond comprehension.

Dr. Daniel Chapin left three sons and three daughters. Col. William Chapin, the eldest, occupied the homestead until his decease, which occurred in 1858. He was much respected. Dr.

Chapin has left with his very few and remaining relatives and friends an odor of pleasant remembrance. I the more readily pay this earnest, if imperfect, tribute to the character of this more than ordinary man for the reason that there existed a reciprocal feeling of respect and confidence between him and the subject of this paper.

After Dr. Chapin was incapacitated for the practice of his profession, he was accustomed to recommend Dr. Trowbridge to his relatives and patients. Between Drs. Daniel and Cyrenius Chapin there always prevailed an intense but wordy rivalry. In some respects they resembled each other, especially in the possession of an indomitable will. The professional differences of these two veterans were always the source of much amusement to their friends, and there were many characteristic anecdotes related of their encounters.

From Mr. Ketchum's book, entitled, "Buffalo and the Senecas," I am enabled to glean a few items relating to Doctor Ebenezer Johnson: He studied his profession in Cherry Valley, under the direction of the celebrated Doctor White; he arrived in Buffalo in 1809; was surgeon on this frontier during the war, and continued an active member of the county society until 1821; established a drug store; was associated with Judge Wilkeson; subsequently became a banker and broker, accumulating a handsome property; was elected the first mayor. In common with others, he had to meet the business revulsion of the times, losing his all. He then emigrated to the State of Tennessee, where, after a residence of a few years, he died.

As considerable time has elapsed without producing any fitting tribute to the memory of one whose civil and professional life always exhibited the highest virtues that should adorn a man, I am constrained by the suggestion of his few early and remaining friends and professional associates, and a personal sense of duty, to assume what to me is a delightful, if it is a delicate task, namely, to prepare a brief memoir of my father, Doctor Josiah Trowbridge.

The subject of this paper was descended from a highly respected English family. He was born in Framingham, Massachusetts, September 28th, 1785. His American ancestry occupied an elevated position among the early colonists. He was a descendant of Thomas Trowbridge, a gentleman of means, who emigrated to this country in 1636. The name of Trowbridge first appears in Doomsday Book. A younger branch of the Devonshire family of Trowbridge seems to have settled in Somersetshire as early as 1541. They resided at Taunton, in that county. From this branch sprang the Trowbridges of America. That the Taunton family descended from that of Devonshire is sufficiently proved by their arms being precisely the same as those seen in the stained glass window in the chancel of St. James' Church, Devonshire.

Among the descendants was Edmund Trowbridge, who graduated at Harvard College at the age of nineteen, and was one of the most learned lawyers in Massachusetts. By his severe toil and discipline he surmounted every obstacle in his honorable career. He was a member of the Colonial Council; appointed Attorney-General of the province, and afterwards Chief Justice; was acknowledged to be a learned man; reckoned preëminent on the bench and at the bar, and is said to have exercised a salutary and elevating influence on the younger members of the profession with whom he associated. Many of the most distinguished lawyers in Massachusetts enjoyed the advantages of his instructions. The eminent jurist, Chief Justice Parsons, was ever ready to acknowledge the benefit he experienced from his early intercourse with Judge Trowbridge.

The father of Doctor Trowbridge was no ordinary man, as the records show. He served in the revolutionary army, first at the battle of Bunker Hill, then as orderly sergeant with Washington, in New Jersey and Pennsylvania. He was with the overland expedition, under the command of Arnold, in his attack on Quebec, and, before leaving the service, I think, was captain; was selectman eight years; town treasurer nineteen years; representative ten years; delegate to the State Convention in 1820; trustee of an academy twenty years, and left, at his death, a handsome legacy for the benefit of indigent students. The record closes by saying that he was held in high esteem.

The mother of Doctor Trowbridge, like all mothers of good sons, was more than an ordinary woman; pious, affectionate, devoted, and making it her daily duty, to cultivate all social and Christian graces in the family. Until the subject of this sketch was somewhat advanced in years he remained at home, assisting his father on the farm. This proving too laborious, and his health not being equal to the task assigned him, in 1799 he took the position of clerk with an elder brother, who was engaged in mercantile pursuits in Boston. Tiring of this, and seized with a desire to see more of the world, he shipped for Holland in 1800, and, after a most tempestuous return voyage, the vessel reached Charleston, South Carolina, in a disabled condition. He returned to his home, and undertook a course of preparatory studies with a view to the adoption of the medical profession. Commencing his readings with Doctor Willard, of Uxbridge, he finished with Doctor Kitteridge. I have frequently heard him mention the names of these gentlemen with respect and evident pleasure.

During this time he taught school two winters, one in Southboro and the next in his native town. He used to relate, in an amusing way, his experiences as school teacher, involving the manner in which he quelled—what was then quite common—rebellion against the teacher. I need not dwell on the experiences of the teacher in those primitive times, further than to refer to the necessity of the strong application of what may be styled military law, when the teacher assumed the only arm of defence at hand, the poker, and disposed of the insurrection as summarily as the necessities of the case required. According to his representations, like the rebellion among us, one determined effort was sufficient to end the struggle. After the usual course of study, he was licensed to practice. This was in 1808 or 1809.

His first professional efforts were put forth in Weathersfield, Vermont, where he remained for a brief time. In company with a young lawyer, an intimate friend, by the name of Walker, in the spring of 1811, he came to Buffalo on horseback. On this journey they made the acquaintance of the distinguished lawyer, Elisha Williams, and of his wife. The latter afterwards spoke of Doctor Trowbridge as her "handsome doctor." Mr. Walker returned to Vermont, where he obtained eminence as a lawyer, and considerable political prominence. Buffalo not offering sufficient encouragement to Doctor Trowbridge, he took up his residence at Fort Erie. At this time that place and Black Rock were the enterpots of the then limited commerce of the lakes, Buffalo Creek being blockaded by sand-bars. Doctor Trowbridge remained in Canada until the declaration of war, when he returned to Buffalo. During his residence in Canada he formed an attachment which, notwithstanding the belligerent condition of the two countries, resulted in a much more than friendly arrangement between himself and one of His Majesty's subjects. I cannot do better than make an extract from a letter, dated October 11th, 1813, written to his brother, in which he says: "I will now inform you something of myself and family, for you must know that I am married. I am not a friend to the war, but I could not forbear engaging in an expedition. On the nineteenth of September, 1813, I crossed into the Province of Upper Canada, and succeeded, with the assistance of Cupid, (who, by the by, is a good general,) in capturing one of His Majesty's subjects, without bloodshed. I have her now in close confinement, and hope I shall be able to keep possession until I have the pleasure of presenting her to you." He was married in Buffalo by the Rev. Elkanah Holmes, a Baptist missionary, September 22d, 1813, to Margaret Wintermute. Before and during the war he was associated with Doctor Cyrenius Chapin, who had preceded him in his location.

As my friend, and the common friend of both these gentlemen, Doctor Pratt, has read to this society a lengthy and interesting paper on Dr. Cyrenius Chapin, it will not be necessary for me to dwell on his character. How long this copartnership continued I have no sufficient data to enable me to say; it commenced before the war and ended directly after its close. His next business connection was with a most estimable man and physician, Doctor John E. Marshall, the first medical man that settled in Chautauqua county, and who, upon its organization as a distinct county, was elected or appointed county clerk. He died in 1838, greatly beloved and respected. The first copartnership was formed September 8th, 1823, under the name of Marshall & Trowbridge. In 1829 the firm was dissolved in consequence of the ill health of Doctor Marshall, he retiring from practice, when the copartnership of Trowbridge and Sprague was formed. October 16th, 1830, Dr. Marshall renewed practice, and the firm of Trowbridge & Marshall was established, and continued until December 2d, 1831. He was an honor to the profession and a useful member of society.

My early remembrances of Doctor Marshall are all pleasant. It would afford me pleasure to dwell more at length on his many agreeable qualities, were it not that I am anticipated by a careful and well-written memoir, prepared by Doctors Josiah Trowbridge and Frank H. Hamilton. Those who desire to know more of Dr. Marshall, I refer to the *Buffalo Medical Journal*, vol. 6th, October, 1850, page 282. Dr. Trowbridge was also associated with Drs. Bela H. Colegrove, Thomas B. Clark and Alden S. Sprague; at a much later period with Dr. Charles Winne. All these gentlemen obtained eminence in their profession and challenged respect as citizens. Dr. Sprague continued to practice his profession in this city, as most of us know, with great success. He died in 1863. I am thus brief, for the reason that there was prepared a biographical notice of the doctor by his nephew, E. C. Sprague, Esq., which appeared at the time in the *Commercial Advertiser*, January 8th, 1863. Dr. Thomas B. Clark, a most estimable and excellent citizen, removed to Detroit, where for a few years he gave his profession his attention, operating, at the same time, in real estate, by means of which he became wealthy. He died in that city, honored and respected.

Dr. Colegrove retired from the firm and returned to the town of Sardinia, where he resides at an advanced age, with accumulated honors derived from his profession. In a recent communication from this venerable man, he informs me that he was born in the State of Rhode Island the twentieth of April, 1799. At the age of eighteen he commenced the study of his profession with Dr. Hubbard, of Pomfret, Connecticut, who, after the death of Prof. Nathan Smith, then occupying the chair of surgery in the New Haven Medical College, was appointed Professor of Surgery in his stead. After attending lectures at New Haven, the University of Pennsylvania, and College of Physicians and Surgeons in New York, he graduated at the latter institution, his diploma being first recorded in the County Clerk's office, of this county, in the year 1821.

He has now been a resident in the town of Sardinia for nearly a half a century, with the exception of the short period he was associated with Marshall and Trowbridge. His rides extending over some portions of this county, Wyoming, Chautauqua, Cattaraugus, and many of the northern counties of Pennsylvania. Is an ardent and industrious man in his profession. There are very few, if any medical men, who have had so extended and varied practice as Doctor Colegrove. He has been ever ready, and eminently capable, to successfully encounter its responsibilities. He says: "I love to "cherish a recollection of my cotemporaries. With the exception "of Doctor Carlos Emmons of Springville, and, perhaps, one or "two others who were in the county of Erie in 1820, all are gone.

"Among the departed, I remember no one in whose character "was blended high professional attainments, with a nicer apprecia-"tion and practice of those courtesies and amenities, personal and "professional, which made the green oasis in human life, than your "father. While you, my dear Doctor, with a filial affection in the "highest degree commendable, are weaving a deserved garland for "the brow of your late excellent father, permit me to add my hum-"ble chaplet to the wreath; which, though the product of an obscure "mountain town, is no exotic: and while the younger members of "our profession are seeking models to emulate, and imitate,—sure "I am, they will seek in vain for a better than the late Josiah "Trowbridge." It affords me pleasure to be able to testify that these kind expressions of respect and affection were reciprocal. Doctor Charles Winne you have with you, and propriety forbids me to speak of him as kindly as my feelings prompt me.

During the war of 1812, Doctor Trowbridge gave the government his hearty support, although, in common with a large political party, not recognizing its absolute necessity. He was at no time in the regular service, but was attached to a volunteer company of artillery, and always ready to respond when humanity or the interests of his country made a demand upon him. After the close of the war he received a grant of land for his services, in the State of Indiana. He was fond of the gun, and on one occasion, in company with Frederick B. Merril, Lieut. Dudley, of Perry's fleet, and one or more boatmen, while shooting ducks on Strawberry Island, was surprised and captured by the British and taken to Fort George. While there, the Indians in the service of the government broke into their quarters, and for a time their safety seemed quite precarious. The interposition of officers and chiefs prevented a massacre. The Doctor with his comrades was detained a few days and then discharged, arriving in Buffalo after a most tedious journey on foot.

During the war he, with most of the early settlers, had many adventures, but none of sufficient importance to occupy your time. At the burning of the village of Buffalo, which was a surprise, although it had been threatened, he, with others, remained engaged in securing the safety of the women and children, and was among the last to leave. As he passed up Main street, he was fired upon by the Indians ambuscaded in the vicinity. With other refugees, he spent the winter, and part of the spring, at various localities east of Buffalo.

Among his experiences at this time was one at the boarding house established by the erratic John Root. Perhaps the credit of this establishment belonged more properly to Mrs. Root. It was at a locality then known as "Harris Hill," three or four miles beyond Williamsville, a small log house, containing one room on the first floor, answering for parlor, dining room, kitchen, &c. The second story was reached by a ladder, furnishing a commodious sleeping room, divided by blankets, for Mr. and Mrs. Heacock, Doctor Trowbridge and wife, and Stephen K. Grosvenor. I only regret that the present opportunity is not the one to revive something of these early and prominent persons and pioneers.

The testimony of Mrs. R. B. Heacock is, that Mr. Root was more successful in supplying his individual wants, than he was the table and the house. Since the preparation of this paper was commenced, this venerable Christian and eminently charitable woman, has been gathered to her heavenly home. Doctor Trowbridge was absent one winter in Washington, on private business and business connected with the interests of the village. In the course of a long life he was an applicant for office in but two instances. Early in life for the position of Collector of Customs, when he was induced to make way for Major Dox, and much later in life for the Post Office.

He continued to practice his profession until 1836, when he had accumulated a handsome property. He then sold his office property to Doctor Winne, and gave his time to the management of his private affairs—attending to the improvement of his property, erecting the United States Hotel and other buildings, and loaning his name and money among his supposed friends. In 1837, when all went by the board, he was unfortunately included among them, losing his all. He made an assignment and was left penniless. This year he was elected Mayor, Hon. Bela D. Coe, an old and intimate friend, being the opposing candidate.

The Patriot war occurring shortly after he was installed, law and order being put at defiance, and he being unable to maintain the supremacy of the former, resigned, and was succeeded by P. A. Barker. During the years described in these sheets, Dr. Trowbridge held many minor places of usefulness. Once, for a short time, he was side Judge; several times he was Supervisor; was associated with Maj. John G. Camp, as Commissioners for finishing the building now known as the "Old Court House." He was President of the High School, afterwards known as the "Buffalo Military, Scientific and Literary Academy." He was Secretary of the meeting held at the house of Elias Ransom, for the purpose of organizing the Episcopal Society of St. Paul's Church, February 10, 1817. He was one of its first vestrymen, held the position for eleven years, and was warden for six. The original church edifice was enlarged in 1828, under a contract entered into by him, at an expense of twenty-five hundred ($2,500) dollars, he depending upon the increased sale of sittings to make good the outlay. It proved to be a remunerative experiment for the parish. He was the last survivor of the original number—was always greatly interested in the prosperity of the church, giving his services to its members and contributing, frequently beyond his means to its treasury.

I will state, in this connection, that he was the friend and medical adviser of Joseph Ellicott, agent of the "Holland Purchase," and through his influence Mr. Ellicott was induced to deed, for church purposes, the lot now occupied by St. Paul's Cathedral. The Presbyterian society taking the hint, made application and obtained the opposite lot. In the year 1839, he was appointed a Commissioner to represent certain rights possessed by the State of Massachusetts in the lands owned and occupied by the Seneca tribe of Indians, about to be ceded to the "Ogden Land Company" by treaty and purchase ; but, for reasons never explained, he was removed. Perhaps the real reason was, that he expressed an opinion that it would be better for the Indian and the white man, were they more distinctly separated.

There were great differences of opinion as to the means employed in the consummation of this treaty. All interested entertained and expressed their own opinions, and in the arguments the reputation of both sides suffered materially, if the various representations are to be believed. As to the merits of these differences, Doctor Trowbridge was never an interested party. It was his opinion that it would prove an expensive purchase to the company, and a favorable treaty for the Indians.

He was actively engaged in his profession in this locality about fifty years, with only one interruption. His rides extended over long distances without reference to day or night, the weather, or circumstances as against personal comfort. Their bounds included Cattaraugus Creek, Olean, Batavia, Lockport, Tonawanda, Lewiston and Canada; late in life he was called as far as Detroit. In 1838 he resumed his profession with Doctor Charles Winne, and continued until 1842. During a portion of this time the writer was a student in their office. Having completed his studies he was associated with his father, continuing until the fall of 1852.

Doctor Trowbridge did a large private and consultation practice until 1856, when increasing infirmities compelled him to relinquish it entirely.

I have forgotten to mention, in its proper order, that in 1833, the honorary degree of "Doctor of Medicine," was conferred upon him by the "Regents of the University."

Thus you have the incidents conneeted with a long and unpretend ing, but eminently practical and useful life.

His excellencies were appreciated and most intimately known among suffering humanity, or in quiet association with his fellow men. He never aspired, by means practiced by many, to be known as a popular man.

Those who committed themselves to his care were not subject to doubtful experiments with a view to his personal aggrandizement. He exhibited all those qualities that are needful to constitute the true gentleman, and his amenities were extended to all without distinction.

I never knew him, by word or act, to depart from strict honor and decorum. His life was that of one who, in his intercourse with men, exhibited all personal virtues and generous sentiments, founded on an intelligent Christian faith, which is necessary to the good citizen and true physician. In his associations with his medical brethren, it was his highest aim to merit their confidence, and without reservation to give them such aid and comfort as the necessities of the case would seem to require. I think I can safely state that in but few instances was this confidence misplaced. The younger members of the profession, with whom he came in contact, were always treated with the most careful consideration. Errors were quietly indicated, and the proper method for their rectification pointed out. We all have had our youthful hopes and aspirations brought low, and our pride grievously wounded by the unfair, overbearing and supercilious treatment received from our seniors, to whom we looked not only for professional assistance, but for that material aid to which the young practitioner is entitled; but pride was never wounded by him. With Doctor Trowbridge, honor was the polar star. In the early organization of society there was no lack of men of enterprise, possessing high moral character, but with them there were mingled the usual proportion of hardened individuals, lacking principle, but still influential through native talent and energy. Our profession had its proportion. Profanity and intemperance were, unfortunately, lightly regarded, in the cases of both the physician and the citizen. The parties themselves were not entirely responsible for this unfortunate condition of morals. The practices of society tended to make drunkards, not only of medical men, but of all—not excluding woman. With the primitive doctor it seemed essential in his exposures that he should fortify his inner man with stimulus; as often as he visited a patient, it was thought necessary that he should take something internally, that the external man might be protected. As a consequence a popular practitioner, with a large field of operations, could hardly fail to be more or less under the influence of stimulants before the day's work was ended.

There was no discredit in reasonable intemperance. It was a common saying that "if you can find a sober, you will find a more than ordinary doctor." To such temptations were he and all exposed; the only matter of wonderment is that any escaped these direful influences. Notwithstanding this condition of society Dr. Trowbridge not entirely unaccustomed to the practices of the day, ignored the use of alcohol, and to this rule he most religiously and steadily adhered. It was only near the close of his life, and upon the recommendation of a medical friend, that he consented to use a certain and exact quantity of stimulus daily. I mention this for the reason that it occurred long before the temperance movement was attempted, or had obtained any influence on the popular mind, when any endeavor to practice temperance was rather a cause of reproach than merit.

In his business habits Dr. Trowbridge was negligent in securing his just dues, though punctillious in the discharge of his personal liabilities. He was never exorbitant in his charges, seldom if ever employing the law in the enforcement of his claims, and often making liberal deductions, or donating his services. The consequence was that, notwithstanding a large practice, he was often pressed for means. With his friends he always erred on the side of liberality. His purse was never closed to the demands of religion and charity. He had many pupils,—among them I name Drs. Frank B. Ransom, Charles C. Haddock, O. S. St. John, Badger, Dunn, and James P. White. The number and the positions obtained in their profession and society indicate his ability as a teacher. Those living will bear me out when I assert he always endeavored to influence them with high and honorable sentiments, not only his teachings but by his example. By some he was preceded to the grave, while others remain to testify to his many excellent qualities. It is not to the discredit of his associates to say that he was a courageous, safe and judicious practitioner. He was constitutionally, and otherwise, eminently calculated for the practice of his chosen profession. In the exercise of its high and often unexpected and exhausting responsibilities, and in all the varying circumstances and vicissitudes incident thereto, there was no one, with the lights then at command, better schooled or fitted to meet them all than he. In the obstetrical science no one of his time, or perhaps since, in this locality, was or is his superior. He always appreciated the grave responsibilities surrounding him, possessed an intelligence that was equal to any emergency, and professional boldness that was ready for every necessity, the want of which is a serious detriment to the success of the more timid but equally intelligent and conscientious practitioner.

In conversation he was peculiarly happy, entertaining and instructive; and, in whatever principle was not concerned, he yielded ready obedience to social usage and custom. He had a faculty of satisfying his pride—for at heart he was a proud man—by honoring the avocation through which he had achieved the right to be what he was.

His leisure time was either employed in general literature, or in keeping pace with the progress of his profession. Politically he always had the best interests of the country at heart, although rarely or ever taking an active part in politics. Early in life he was a Federalist; later a Whig, and lastly a Republican. The government, in its earlier efforts to maintain itself as against England always had his best efforts, and in its recent and successful attempt to maintain the supremacy of the Union and the Constitution was the recipient of his devoutest wishes. Two of his sons were in the service; one of them, the youngest, Capt. Henry W. Trowbridge, of the 5th Michigan, died while his regiment was in front of Yorktown. Dr. Trowbridge often expressed a desire to live to see the government recover its control over the rebellious States, and to assert and successfully maintain its supremacy, but this was not allowed him.

The religious faith and opinions of one who has satisfactorily finished his earthly mission, is always a delicate subject. It is too much the case that men are appreciated and judged in proportion as they are prospered in the goods of this world. Wealth too often gives its possessor a social position to which his deficiencies in moral and educational qualities do not entitle him. Religious belief and practice are very greatly dependent upon education, circumstances, and the will of the individual. Faith is manifested in various ways—with some it is quiet and unostentatious; with others, demonstrative and presuming. Dr. Trowbridge's parents were Puritans, and as his father's house was his home until he was quite a young man, it is fair to suppose there were no opportunities neglected in attempting to impress on his youthful mind their religious belief. Addison remarks, that these early impressions are never entirely lost, although they may fail in their full fruition.

In conversation Dr. Trowbridge would often refer, pleasantly and with profound respect, to the rigid strictness of his most excellent and pious father, and also with the utmost affection to that of his devoted and pious mother. While clerk in Boston, he attended the only Episcopal church in that city, then known as "King's Chapel," and as his wife was educated in the Episcopal faith, he was thus influenced and confirmed in his early predilections. He was a communicant with this body before my remembrance. The truths and requirements of religion were received and discharged by him with an intelligence worthy of their importance. His belief was earnest, simple, honest, founded in the exercise of the high prerogative of reason. It was neither uncharitable nor sectarian. He was always ready to concede to others the right and the opportunity of an honest christian difference, rebelling most stoutly against pretence and cant, whether it had an existence in religion, morals or the medical profession. The Bible and the Book of Common Prayer, for many years, had a place on his table, and every day were the subject of his study.

In active life his labors on the Sabbath were always arranged, when possible, so that those portions of the day devoted to public worship might not be subject to interruption. Judging him by what makes up the actualities of life, I am safe in claiming for him a reasonable effort to live acceptably to God and man.

Towards the close of his life, and after he was incapacitated for the active duties of his profession, the subject of religion was a matter of serious consideration and frequent conversation. In his expressions he never made place for doubt. The near and certain approach of death was calmly awaited, although he expressed himself as having in an imperfect manner, and, perhaps, not according to the talents committed him, completed his usefulness.

Fearing that he might be regarded as a burden, he was ready and prepared to give place to others, physically and otherwise, better prepared to accomplish the never-ending duties of life, only expressing a desire to be exempted from that intense physical suffering which is often attendant upon the final departure of the soul. In this not unreasonable request he was Providentially gratified. It was my practice to spend a portion of each of his latter days in his company. I left him on the evening of the twenty-seventh September, 1862, in his usual health and spirits. Early the next morning I was suddenly but not unexpectedly called to find him in an insensible condition. He rallied for a short time, not sufficiently long to be able to give expression to his wishes, but so as to be conscious of his situation. Soon, with a smile on his countenance, and without pain, he passed into that eternity in which we hope and believe the cares and distractions of life and the necessities of administering to the physical sufferings of humanity are ended.

The public, by private manifestations, the press, the medical profession, each in their own proper way, gave expression to the loss that society had been called upon to sustain. This was the last of a long and useful life. If there is purity here below, then it was pure.

---

The foregoing paper was read at the Annual Meeting of the Erie County Medical Society, January 12th, 1869:

*Resolved*, That one thousand copies be printed, and that six hundred be bound in the "Buffalo Medical Journal."

And also read before the "Buffalo Historical Society," January 19th, 1869.

